# Current trends in breast cancer genetics, risk factors, and screening strategies

**DOI:** 10.14440/jbm.2025.0079

**Published:** 2025-03-13

**Authors:** Sorana Caterina Anton, Alexandra Lazan, Mihaela Grigore, Ciprian Ilea, Şadiye-Ioana Scripcariu, Setalia Popa, Simona Volovăț, Bogdan Doroftei, Delia Nicolaiciuc, Diana Popovici, Gabriel Costăchescu, Ovidiu Sebastian Nicolaiciuc, Emil Anton

**Affiliations:** 1 Department of Obstetrics and Gynecology, Faculty of Medicine, University of Medicine and Pharmacy “Grigore T. Popa,” Iaşi 700115, Romania; 2 Department of Medical Genetics, Faculty of Medicine, University of Medicine and Pharmacy “Grigore T. Popa,” Iaşi 700115, Romania; 3 Department of Medical Oncology, Faculty of Medicine, University of Medicine and Pharmacy “Grigore T. Popa,” Iaşi 700115, Romania; 4 Department of Preventive Medicine and Interdisciplinarity, Faculty of Medicine, University of Medicine and Pharmacy “Grigore T. Popa,” Iaşi 700115, Romania; 5 Department of Implantology, Removable Restorations, and Technology, Faculty of Dental Medicine, University of Medicine and Pharmacy “Grigore T. Popa,” Iaşi 700115, Romania

**Keywords:** Breast cancer, Breast cancer screening, Risk factors, Breast genetics, Prognostic markers

## Abstract

**Background:**

Breast cancer is the most frequently diagnosed female malignancy worldwide and one of the primary causes of cancer-related mortality in women. According to the latest the World Health Organization data, it was the most common cancer in 157 out of 185 countries and was culpable for an estimated 670,000 deaths in 2023. With breast cancer incidence continuing to increase, there is a mounting interest in early detection and prevention, with a focus particularly directed on genetic factors, modifiable risk factors, and screening methods.

**Objective:**

This review aimed to examine the genetic landscape of breast cancer, the role of risk factors in disease development, and the importance of advancing diagnostic modalities for early detection. A comprehensive search and analysis of peer-reviewed articles and clinical studies from major medical databases were conducted to assess the most recent advancements and discoveries in the field. The literature review identified several modifiable and non-modifiable risk factors, including genetic predispositions (e.g., *BRCA* mutations), hormonal influences, lifestyle factors, and reproductive patterns.

**Conclusion:**

By synthesizing current knowledge, this review enhances the understanding of breast cancer’s multifactorial nature and provides insights to guide future research on screening strategies and preventive measures.

## 1. Introduction

Carcinogenesis has the potential to occur in every cell, tissue, and organ, resulting in pathological changes that give rise to an extensive array of malignancies. The primary mechanisms driving its progression include metastasis, unlimited proliferative capacity, enhanced angiogenesis, induction of self-sustaining growth signals, resistance to growth-inhibitory signals, and evasion of apoptosis.^1^ Breast cancer represents the most common cancer among women across the globe, accounting for 11.7% of all cancers worldwide in 2020.^2^ Over the past two decades, its incidence has increased significantly, especially in low- and middle-income countries, although the absolute incidence remains lower than in high-income countries.^3^,^4^ In the United States, the incidence of breast cancer is rising by approximately 0.6%/year.^5^ In 2020, virtually 685,000 global deaths were attributed to breast cancer.^1^,^6^

A systematic literature search was conducted using the following keywords: “Breast cancer,” “Breast cancer screening,” “Risk factors,” “Breast genetics,” and “Prognostic.” We searched PubMed, Medline, Science Direct, and clinical trial registers (www.medresman.org), as well as websites of major organizations (e.g., the International Association of Breast Cancer and the American Society of Breast Cancer for Women). Literature published between 2013 and 2025 was reviewed with an aim of consolidating essential information on breast cancer risk factors to support prophylactic strategies for at-risk populations.

Studies were included if they focused on the relationship between risk factors, genetics, and breast cancer, as well as screening modalities. Only articles with accessible full texts and comprehensive data presentation were considered. Exclusion criteria included non-peer-reviewed articles, opinion pieces, editorials, case reports, and conference abstracts. Studies having incomplete data, lacking methodological rigor, or raising ethical concerns were also excluded, along with articles available only in abstract form without full-text access.

Breast cancer comprises a heterogeneous group of tumors classified based on histological type, tumor pattern, and molecular characteristics. These classifications are crucial for predicting prognosis and guiding treatment decisions. Current treatment options for breast cancer include surgery, radiation therapy (external and internal), chemotherapy, hormone therapy, targeted therapy (e.g., monoclonal antibodies, tyrosine kinase inhibitors, cyclin-dependent kinase inhibitors, mammalian target of rapamycin inhibitors, and poly-ADP ribose polymerase inhibitors), and immunotherapy.^7^ Surgical management of breast cancer varies depending on tumor size, location, and disease stage. The main surgical procedures include lumpectomy, which preserves the breast while removing the tumor with or without lymph node excision; total mastectomy, which involves the removal of the entire affected breast with or without lymph node removal; and modified radical mastectomy, which entails the resection of the entire affected breast, axillary lymph nodes, nipple, areola, and overlying skin.

Breast cancer originates in breast cells, where malignant tumors, composed of cancerous cells, possess the capacity to invade and destroy adjacent tissues. In some cases, breast cells undergo aberrant changes that impede their normal development or function. These alterations may lead to non-cancerous breast conditions, including atypical hyperplasia and cysts, or benign lesions, such as intraductal papillomas.^8^ The most common site of breast cancer development is the ductal epithelium, whose cancerous change leads to a cancer subtype referred to as ductal carcinoma. Cancer may also arise from lobular epithelial cells, which are organized into the milk-producing glands and might experience cancerous changes classified as lobular carcinoma.

Both ductal and lobular carcinomas can be categorized as *in situ* or invasive.^9^ Less common variants of breast cancer include triple-negative breast cancer, inflammatory breast cancer, and Paget’s disease of the breast, while soft-tissue sarcoma and non-Hodgkin lymphoma represent rare forms of breast cancer.^10^

Among all cases of invasive breast carcinoma, 80 – 85% fall into the type of invasive ductal carcinomas, while 5 – 15% are of invasive lobular type. The remaining cases have other special subtype carcinomas.^11^ Molecular classification immunohistochemically categorizes breast cancer into four subtypes in terms of the expression of hormone receptors: estrogen receptor-positive (ER+), progesterone receptor-positive (PR+), human epidermal growth factor receptor 2-positive (HER2+), and triple-negative breast cancer.^12^

Breast cancer arises as a result of the interplay of multiple internal and external factors.^13^ It is estimated that genetic mutations and familial history account for 5 – 10% of breast cancer cases, while 20 – 30% are attributable to modifiable risk factors.^14^

## 2. Risk factors

The risk of developing breast cancer is influenced by exposure to a variety of factors throughout the life course, including early-life exposures from infancy and adolescence. Many of the known risk factors are summarized in Table 1; however, additional factors require further clarification through well-conducted research.

**Table 1:** Modifiable and non-modifiable risk factors

Risk factor type	Examples
Modifiable	Diet, exposure to artificial light and chemicals, alcohol consumption and smoking, anthropometrics, exogenous hormone therapy, traffic-related air pollution
Non-modifiable	Age, gender, endogenous hormones, ethnicity, economic status, familial and reproductive history, radiation therapy, breast tissue density

### 2.1. Non-modifiable factors

#### 2.1.1. Gender

Women account for the overwhelming majority of breast cancer cases, comprising over 99%, while men represent only approximately 1% of cases.^15^ Unlike men, whose estrogen levels are negligible, women have breast cells that are highly susceptible to hormonal fluctuations, particularly estrogen and progesterone. Any disturbances in the equilibrium of these hormones can contribute to an elevated risk of breast cancer. Elevated levels of circulating estrogens and androgens have been found to be positively correlated with breast cancer risk.^16^ The Endogenous Hormones and Breast Cancer Collaborative Group has further confirmed that alterations in physiological levels of endogenous sex hormones contribute to a higher risk of breast cancer in both premenopausal and postmenopausal women.^17^

In men, several factors significantly increase the risk of breast cancer, including *BRCA2* and *BRCA1* mutations, advanced age, Klinefelter syndrome, elevated estrogen levels, radiation exposure, and a family history of breast cancer.^18^

#### 2.1.2. Endogenous hormones

Sex hormones are essential in breast cancer development, with postmenopausal women being at higher risk due to elevated levels of estrogens, androgens, and prolactin in their bloodstream.^19^,^20^ While estrogen metabolites may also be contributors to breast cancer risk, current evidence remains limited.^21^ Elevated levels of anti-Müllerian hormone have been associated with an increased risk of breast cancer, whereas higher concentrations of sex hormone-binding globulin may be a protective factor.^22^ The challenge of accurately characterizing long-term hormone levels may explain why progesterone levels have not been consistently associated with premenopausal breast cancer. Furthermore, insulin-like growth factor-1 has been marginally linked to an increased risk of ER+ breast cancer.^23^ Other circulating biomarkers, such as insulin, leptin, and C-peptide, may also influence breast cancer etiology.^24^

#### 2.1.3. Age

Approximately 80% of breast cancer cases, including triple-negative breast cancer, are diagnosed in individuals over 50, with a particularly elevated risk observed among those aged 40, 50, and 70. Triple-negative breast cancer is more prevalent in individuals under 40, whereas luminal A subtype breast cancer is more common in older patients.^25^

Although rare, young women are more frequently diagnosed with the basal-like molecular subtype, characterized by frequent HER-2 receptor overexpression and minimal steroid receptor expression.^26^ The most recent data from 2021 indicated that, between 2017 and 2021, the median age of breast cancer diagnosis among women in the United States was 63 years. However, this median age varies by race and ethnicity, ranging from 65 years for non-Hispanic white women to 58 years for Hispanic women.^27^

#### 2.1.4. Ethnicity

Racial and ethnic disparities in breast cancer incidence and mortality rates persist, with non-Hispanic white women experiencing the highest incidence rates, while Black women have higher mortality and the lowest survival rates.^28^ Several studies have explored potential explanations for these disparities, identifying differences in the prevalence of specific breast cancer subtypes across racial and ethnic groups. For example, triple-negative breast cancer is practically twice as common in Black women as in their White counterparts. Between 2010 and 2019, the incidence rate of TNBC in the United States was 33.8 cases/100,000 Black women, compared to 17.5 cases/100,000 White women.^29^ Breastfeeding has been identified as a protective factor against triple-negative breast cancer,^30^ yet Black women have lower breastfeeding rates than White women,^31^ which may contribute to this disparity.

Some researchers suggest that racial differences in breast cancer incidence and outcomes may be partially explained by biological variations, including differences in plasma levels of sex hormones.^32^

#### 2.1.5. Economic status

**Figure 1:**
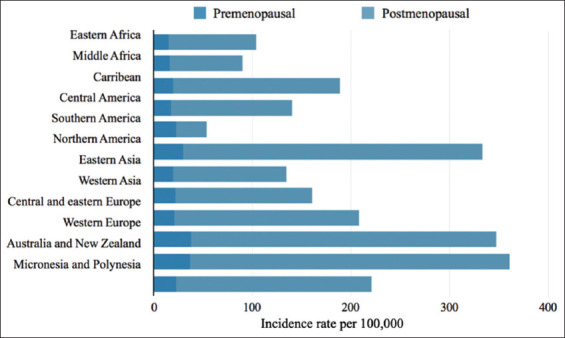
Estimated incidence of breast cancer in 2018 among females of all ages

The global prevalence of breast cancer is rising due to population aging and growth, with industrialized nations exhibiting the highest incidence rates (Figure 1).^33^,^34^

However, in emerging nations, improved access to preventive services, reductions in maternal, newborn, and child mortality rates, and expanded public healthcare coverage have contributed to lower morbidity rates. Despite the lower incidence, breast cancer mortality rates remain higher in low- and middle-income nations.^34^

#### 2.1.6. Familial and reproductive history

A family history of breast cancer is a significant risk factor, with 13 – 19% of breast cancer patients reporting a first-degree relative (mother, sister, or daughter) with the disease.^35^ Individuals with a first-degree relative diagnosed with breast cancer before the age of 40 face a 14.1% increased risk of developing the disease within the next 10 years.^36^ This risk is further elevated in women carrying predisposing mutations in the *BRCA1* or *BRCA2* genes. The presence of multiple affected relatives, especially those diagnosed before the age of 50, further increases the likelihood of disease occurrence. These findings highlight the hereditary component of breast cancer, underscoring the role of shared environmental risk factors, lifestyle influences, and epigenetic modifications. Understanding familial aggregation is crucial for risk assessment, early detection, and the development of targeted prevention strategies. In addition, a family history of ovarian cancer, particularly in cases involving *BRCA1* or *BRCA2* mutations, is also a recognized risk factor for breast cancer.^37^,^38^

There is strong evidence linking hormone exposure levels to breast cancer risk in women. The breast microenvironment may be particularly susceptible to carcinogenic events due to prolonged hormonal fluctuations associated with pregnancy, lactation, menarche, and menopause. Certain reproductive factors have been found to modulate breast cancer risk. For instance, early full-term pregnancy and pre-eclampsia exert a protective effect against breast carcinogenesis. Hormonal dysregulation during pre-eclampsia, characterized by increased progesterone and decreased estrogen levels, further contributes to this protective effect. In addition, protracted lactation and an earlier age at menarche have been associated with a reduced risk of breast cancer.^39^

#### 2.1.7. Radiation therapy

Individuals who have undergone radiotherapy before the age of 30 are at an increased risk of developing breast cancer, with susceptibility influenced by factors such as individual characteristics and age at exposure.^40^ The risk of secondary malignancy can be minimized through the use of optimized radiotherapy techniques, such as tangential field intensity-modulated radiation therapy. However, a family history of breast cancer may further elevate the risk, while incorporating supplementary radiation into conventional radiotherapy protocols has been shown to lower local recurrence rates.^41^

#### 2.1.8. Breast tissue density

Extremely dense breast tissue is associated with a one- to six-fold increased risk of breast cancer, with mammographic density recognized as a well-defined risk factor. In comparison to women with Breast Imaging Reporting & Data System (BI-RADS) density B, those with BI-RADS density D are subject to an approximately twofold higher risk of breast cancer.^42^

In general, breast cancer risk is positively correlated with breast tissue density, a trend observed in both premenopausal and postmenopausal females. It has been suggested that assessing breast tissue density may serve as a rapid, non-invasive tool for identifying women at elevated risk, thereby facilitating enhanced monitoring and early detection strategies.^43^

### 2.2. Modifiable factors

#### 2.2.1. Diet

Certain dietary patterns have been associated with breast cancer risk. Consumption of processed meats has been linked to an increased risk, whereas low-fat dairy, fruits, and vegetables appear to have a protective effect.^44^ The relationship between coffee and tea consumption and breast cancer risk remains inconsistent across different subgroups.

Several nutrients exhibit protective properties against breast cancer. Calcium, Vitamin D, and carotenoids have been shown to reduce risk, whereas increased heme iron intake and elevated plasma iron levels may contribute to a higher risk.^45^ The majority of other nutrients, such as Vitamins A, B vitamins, Vitamin C, Vitamin E, folate, selenium, phytoestrogens, and isoflavones, have demonstrated either inconsistent or no significant associations with breast cancer risk. In addition, multivitamin use has not been linked to an increased risk of breast cancer.

Among dietary factors, alcohol consumption bears the most consistent association with breast cancer risk, with a modest increase in risk observed.^46^-^48^ Studies indicate that every 10% increase in ultra-processed food consumption is associated with an 11% increase in breast cancer risk. A diet rich in n-3 polyunsaturated fatty acids, Vitamin D, fiber, folate, and phytoestrogen may help reduce breast cancer risk, while n-6 polyunsaturated fatty acids should be consumed in moderation. In addition, research suggests that curcuminoids (derived from turmeric), green tea polyphenols, and sulforaphane may possess anticancer properties similar to those observed in breast cancer studies.^49^,^50^

#### 2.2.2. Exposure to artificial light and chemicals

Exposure to artificial light at night (ALAN) has been associated with an elevated risk of breast cancer, potentially due to disruptions in melatonin production and epigenetic modifications. Research indicates that individuals with higher ALAN exposure have a greater risk of breast cancer compared to those with lower exposure. In addition, urinary levels of 6-sulfatoxymelatonin, the primary melatonin metabolite, have been correlated with breast cancer risk, likely influenced by light exposure at night or shift work.^24^,^51^

Melatonin, predominantly produced in the pineal gland, is regulated by noradrenergic activity during nighttime. Beyond its role in regulating gonadal activity, nociception, blood pressure, and inflammation, melatonin exhibits anticarcinogenic properties.^52^ Night shift work disrupts circadian rhythms, leading to the suppression of melatonin synthesis and secretion in the pineal gland, which may contribute to an increased risk of breast cancer.^53^,^54^ Even low-intensity nighttime light exposure can suppress nocturnal melatonin production, thereby increasing cancer risk, especially breast cancer in women.^55^ Recognizing this, Denmark became the first country in 2007 to provide government compensation to women who developed breast cancer after prolonged night shift work. Furthermore, a study by Schernhammer *et al*.^56^ found that women engaged in night shift work carried a 1.58-fold increased risk of developing breast cancer compared to those who worked only during the day.

Protracted exposure to certain environmental chemicals may also contribute to breast cancer development by altering the tumor microenvironment, promoting pro-carcinogenic processes, and inducing epigenetic changes. The duration of exposure is a significant factor in determining the increased risk of breast cancer in women exposed to these substances.^57^ Early exposure to pesticides, such as dichlorodiphenyltrichloroethane (commonly found in vegetables, meat, fish, and dairy products), has been linked to disruptions in mammary gland development. Similarly, polychlorinated biphenyls (PCB), a group of synthetic chemicals widely used in electrical equipment (e.g., capacitors and transformers), have been associated with an increased risk of breast cancer. PCBs are present in animal fats and can be ingested through dietary sources (notably catfish, buffalo fish, and carp) or absorbed through inhalation and dermal contact.

In addition, polycyclic aromatic hydrocarbons (byproducts of the combustion of organic materials) are found in cooked foods, synthetic fibers, organic solvents, and oil mist, and their exposure may be associated with a heightened risk of breast cancer.^58^

#### 2.2.3. Alcohol consumption and smoking

Excessive alcohol intake has been associated with an increased risk of breast cancer, primarily due to elevated estrogen levels and hormonal imbalances. Moderate alcohol consumption has been specifically linked to a higher incidence of ER+ breast cancer.^59^

Alcohol consumption often leads to an increase in body mass index (BMI) and excessive weight gain, further compounding breast cancer risk.^60^ Several hypotheses suggest that alcohol may contribute to ER+ breast tumor development by impairing nutritional intake. In 2018, the World Cancer Research Fund and the American Institute for Cancer Research conducted a meta-analysis of 16 prospective studies on premenopausal breast cancer and 34 studies on postmenopausal breast cancer. Their findings indicated that, for every additional 10 g of alcohol consumed per day, the risk of breast cancer increased by 5% in premenopausal women and by 9% in postmenopausal women.^61^ According to the National Institute on Alcohol Abuse and Alcoholism, a “standard” drink contains approximately 14 g of pure alcohol, equivalent to about 148 mL of wine (typically 12% alcohol by volume).^62^ Studies from several European countries, such as Italy,^63^ France,^64^ and the United Kingdom,^65^ have also reported an association between alcohol consumption and an increased risk of breast cancer in women. These findings underscore the significance of alcohol consumption as a public health concern, reinforcing the importance of reducing daily intake to mitigate risk.

The epidemiological evidence regarding smoking as a risk factor for breast cancer has been inconsistent over time. However, recent studies suggest a modest association. Women who are current smokers or have smoked for at least 10 years have an approximately 10% increased risk of developing breast cancer.^66^-^68^ The transport of tobacco-derived carcinogenic chemicals to breast tissue is believed to contribute to oncogene and tumor suppressor gene alterations, especially involving *TP53*. Both active and passive smoking have been implicated in pro-carcinogenic events. Among women with a family history of breast cancer, smoking before full-term pregnancy and a longer smoking history have been associated with a higher risk compared to smoking initiated after pregnancy.^67^ These findings underline smoking as a significant public health concern.

#### 2.2.4. Anthropometrics

Throughout life, body size plays a critical role in the complex relationship between obesity and breast cancer risk. A higher BMI during childhood or early adulthood has been associated with a lower risk of breast cancer, whereas a greater birth weight is linked to a slightly increased risk in adulthood.^69^ In premenopausal women, higher adult BMI appears to have an inverse relationship with breast cancer risk, whereas in postmenopausal women, increased BMI is positively associated with the risk, especially in cases with ER+ tumors and among those who have never used hormone treatment. These differences are hypothesized to result from variations in estrogen levels and their primary sources across menopausal states.^70^ Regardless of menopausal status, a polygenic risk score for adult BMI has been inversely linked to breast cancer risk, as demonstrated in a large Mendelian randomization trial. Recently, Huang *et al*.^71^ identified a significant correlation between breast cancer prevalence and the weight-adjusted waist index, a novel measure of central obesity. Their findings suggest that this index outperforms conventional obesity indicators such as BMI in predicting breast cancer risk.

In addition to BMI, other non-modifiable anthropometric factors, such as height and birth length, have been associated with an increased risk of breast cancer.^72^ Furthermore, high breast density, as assessed by radiological imaging, significantly elevates breast cancer risk, independent of menopausal status or hormone receptor profile.^73^

#### 2.2.5. Exogenous hormone therapy

Several studies have shown that oral contraceptive use is associated with an increased risk of breast cancer, with this risk lingering for up to 10 years after cessation of use. A meta-analysis conducted by Gierisch *et al*.^74^ estimated that lifetime use of oral contraceptives increases the absolute risk of breast cancer by approximately 0.89%. This effect is most pronounced among current and former users. According to the Collaborative Group on Hormonal Factors in Breast Cancer, the increased risk is no longer detectable 10 years after discontinuation of oral contraceptive use.^75^ The effect of oral contraceptive use in women with a family history of breast cancer remains controversial due to limited studies, low statistical power, and variability in the definition of a family history of breast cancer.^76^ Given the widespread use and efficacy of oral contraceptives, clarifying their safety in this population remains a crucial research priority. In addition, levonorgestrel-releasing intrauterine devices have been linked to an increased risk of breast cancer.^77^ Postmenopausal hormone therapy in combination with estrogen and progestin has been strongly associated with an increased risk of breast cancer, particularly among current or recent users and those with prolonged use. However, few studies have examined the impact of different dosages, formulations, and evolving patterns of hormone therapy use.^78^

#### 2.2.6. Traffic-related air pollution

Traffic-related air pollution has been classified as a human carcinogen due to its well-established association with lung cancer.^79^ Recent studies have also identified an association between air pollution – particularly exposure to nitrogen dioxide (NO_2_) and ambient fine particulate matter with diameter ≤2.5 μm (PM2.5) – and an increased risk of breast cancer.^80^,^81^ Although long-term policies aimed at reducing air pollution have been implemented in many countries, more than half of the global population remains exposed to elevated levels of air pollution.^82^,^83^ For every 10 μg/m^3^ increase in NO_2_, LeMarchand *et al*.^84^ reported a higher incidence of ER+/PR+ breast tumors compared to ER-/PR tumors.

#### 2.2.7. Summary

Our review of modifiable risk factors for breast cancer highlights specific populations that would benefit most from targeted interventions aimed at minimizing or reversing these risks. To reduce the likelihood of developing breast cancer, we particularly encourage women at higher risk to adopt primary prevention measures. These include avoiding tobacco consumption, limiting exposure to exogenous hormones and excessive ionizing radiation, maintaining a healthy weight, engaging in regular physical activity, breastfeeding, minimizing night shift work and additional exposure to artificial light when possible, following a nutrient-rich diet, and reducing alcohol consumption.

## 3. Genetic factors

Genetic factors play a critical role in breast cancer risk and have become a key component of both risk assessment and treatment strategies. Numerous studies have demonstrated associations between germline mutations and an increased risk of breast cancer among affected individuals and their relatives. For example, a Swedish study involving 28,362 women, including all breast cancer patients and randomly selected breast cancer-free women from the Karolinska Mammography Project for Risk Prediction of Breast Cancer, found that female relatives of carriers of protein-truncating variants in certain risk genes had s significantly higher risk of developing breast cancer.^85^

Genetic predisposition influences guidelines for screening, monitoring, preventive care, and treatment in women carrying germline mutations in breast cancer susceptibility genes. Research also suggests that concurrent genetic testing at the time of breast cancer diagnosis, as well as the specific mutation type identified, may impact clinical management decisions.^86^ Identifying patient subgroups with distinct prognoses or therapeutic responses can further guide personalized treatment strategies.

Several genes are linked to an increased risk of breast cancer. Mutations in *BRCA1* and *BRCA2* account for the majority of hereditary breast cancer cases and approximately 5 – 10% of all cases. In addition, mutations in other high-penetrance genes, including *PTEN, TP53, STK11, CDH1*, and *PALB2*, have been linked to elevated breast cancer risk.^87^

Genetic testing, using blood or tissue samples, enables the identification of mutations predisposing individuals to breast cancer. The landscape of molecular testing is ever-evolving, with *BRCA* testing now widely available through commercial and academic reference laboratories. In some countries, testing costs are covered by national health systems.^88^ Although genetic testing can be costly and may cause patient anxiety, multi-gene panels allow for the simultaneous assessment of multiple breast cancer-related mutations.^89^ A thorough assessment of personal and family medical history before testing can help mitigate patient anxiety and optimize cost-effectiveness. While *BRCA1* and *BRCA2* remain the most well-established genes linked to breast cancer risk, restricting testing to these two genes may overlook other clinically relevant mutations.^90^

### 3.1. High- and intermediate-penetrance genes

**Figure 2:**
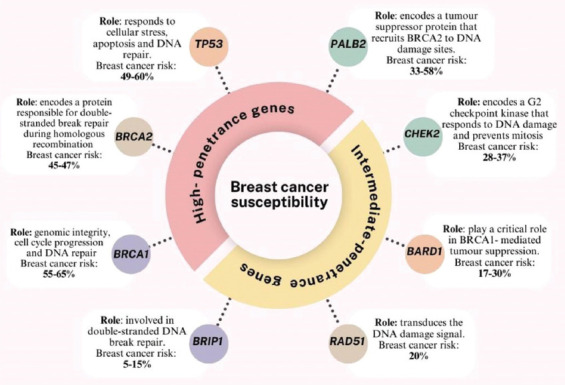
Breast cancer susceptibility genes categorized by penetrance grade and the associated risk for breast cancer in women carrying these gene mutations.^91^-^93^ Figure created by the authors.

It is estimated that approximately 50% of hereditary breast cancer cases are attributed to mutations in high- or intermediate-penetrance genes (Figure 2).

Tumor suppressor genes *BRCA1* and *BRCA2* are essential for maintaining genomic integrity, regulating centrosome dynamics, and ensuring chromosomal, cytokinetic, and genomic stability. Disruption of *BRCA* activity can create a hormone-dependent carcinogenic environment that compromises genomic integrity, generates pro-survival signals, and promotes breast cancer development. In 2004, the term “BRCAness” was coined to describe a phenotype associated with *BRCA1* or *BRCA2* variations, which may serve as a therapeutic biomarker.^94^

The ClinVar database has identified approximately 4,300 distinct germline variants in *BRCA1* and 5,200 in *BRCA2*, categorized as either pathogenic or potentially pathogenic.^95^ Among these, 80% are truncating mutations, leading to frameshift or nonsense changes, often resulting in premature stop codons. In addition, roughly 10% of the identified variants are pathogenic missense variants, while another 10% are attributed to aberrant copy number variations.^96^

Understanding the genotype-phenotype relationship is crucial for assessing the risk of *BRCA1*- and *BRCA2*-associated malignancies. The BRCA Exchange Project aims to establish a comprehensive, international repository of *BRCA* genetic mutation data, providing healthcare professionals, clinicians, and researchers with valuable insights to inform clinical decision-making.^97^

The discovery of additional genes linked to breast and ovarian cancer risk has prompted the development of tumor-associated gene panels and structured frameworks to optimize the management of mutation carriers. Notably, 3.5 – 10.9% of hereditary breast cancer cases involve non-*BRCA1/2* gene variants, which are also significantly associated with breast cancer susceptibility. Eight different genes, including *ATM, BRCA1, BRCA2, CHEK2, PALB2* (*FANCN*), *RAD51C*, and *RAD51D*, demonstrate significant correlations with an increased risk of breast cancer when harboring pathogenic variations.^98^

*TP53* and *CDH1* are examples of high-penetrance genes. Germline mutations in *BRCA1* and *TP53* are predominantly associated with invasive ductal carcinoma, whereas *BRCA2* germline alterations are associated with both ductal and lobular breast cancers. In contrast, *CDH1* germline mutations are specifically associated with lobular breast cancer, especially the invasive subtype, and are strongly correlated with hereditary diffuse gastric cancer syndrome.^99^,^100^

The *PALB2* gene encodes a binding partner of BRCA2. Biallelic mutations in *PALB2* cause Fanconi anemia, while monoallelic *PALB2* mutations are associated with breast and ovarian cancer, carrying an estimated absolute lifetime risk of 41 – 60% and 35%, respectively. Pathogenic PALB2 variants are found in approximately 0.4 – 3.9% of individuals, and those carrying such mutations have a 35% lifetime risk of developing breast cancer by the age of 70.^101^

The tumor suppressor gene *TP53*, located on chromosome 17p13, is important in cell cycle regulation and apoptosis. Germline pathogenic mutations in *TP53* cause Li-Fraumeni syndrome, a rare autosomal dominant disorder characterized by a markedly increased predisposition to malignancies across a broad spectrum of tumor types.^102^ Among women diagnosed as having breast cancer before the age of 31, 3.8 – 7.7% carry disease-causing germline *TP53* mutations. However, the pathogenicity of *TP53* germline mutations varies, depending on the specific molecular alteration, making the identification of key determinants in *TP53*-associated tumorigenesis a major focus of current research.^103^,^104^

The *ATM* gene is essential for DNA double-strand break repair. Germline pathogenic mutations in ATM cause ataxia-telangiectasia, a syndromic disorder characterized by progressive cerebellar ataxia, oculomotor apraxia, immunodeficiency, and an increased risk of malignancy.^105^ Heterozygosity for *ATM* loss-of-function mutations has been implicated in increased cancer susceptibility, though studies suggest that carriers of *ATM* pathogenic mutations do not exhibit a significantly elevated risk of contralateral breast cancer (CBC) compared to non-carriers.^106^

For individuals with pathogenic *ATM* mutations, the relationship between radiation exposure and breast cancer risk remains complicated. The Women’s Environmental Cancer and Radiation Epidemiology project investigates the interplay between radiation exposure, genetic susceptibility, and breast cancer risk – specifically, radiation-induced CBC.^107^ Findings indicate that women with rare, likely pathogenic *ATM* missense variants exhibit a dose-dependent increase in CBC risk, whereas carriers of common *ATM* variants may enjoy a protective effect that reduces CBC risk.^108^

The *BARD1* gene is intrinsically linked to the BRCA1 protein in terms of both structure and function and BRCA1 protein is involved in DNA repair and apoptosis. While germline *BARD1* variants have been associated with an increased risk of breast cancer, the evidence supporting risk-reducing mastectomy for *BARD1* carrier’s remains limited.^109^

The *BRIP1* gene encodes a helicase protein involved in DNA repair, specifically in the repair of interstrand cross-link damage. Although there is no conclusive evidence linking germline *BRIP1* mutations to an elevated risk of breast cancer, these mutations are significantly correlated with an absolute lifetime risk of up to 15% for ovarian cancer. *BRIP1* is the third most frequently linked gene to ovarian cancer susceptibility, with pathogenic variants detected in 0.9 – 2.5% of ovarian cancer patients.^110^

The *CHEK2* gene encodes a tumor-suppressor protein that plays a critical role in DNA repair, cell cycle arrest, and apoptosis in response to DNA damage.^106^ Approximately 1% of individuals of European descent carry a pathogenic *CHEK2* mutation. Several pathogenic variants in *CHEK2* have been identified, such as 1100delC, I157T, R117G, I160 M, and G167R.^111^

The *RAD51C* and *RAD51D* genes, which are paralogs, are implicated in non-homologous end joining, homologous recombination, and double-strand break repair. Pathogenetic variants in *RAD51C* and *RAD51D* are associated with a lifetime breast cancer risk of 20 – 40%.^112^ Current management strategies for individuals carrying these mutations include annual mammography (MAM) and contrast-enhanced magnetic resonance imaging (MRI) screening starting at age 40.

### 3.2. Low-penetrance genes

Approximately 50% of heredity breast cancer cases can be attributed to pathogenic mutations in high- and moderate-penetrance genes. However, a substantial proportion of unexplained heritability is likely due to a combination of common and rare genetic variants with varying frequencies and penetrance.^109^ Over the last 10 years, population-based genetic association studies, including large-scale genome-wide association studies (GWAS), multistage GWAS, and candidate gene association studies, have gained increasing significance. GWAS has identified over 180 single-nucleotide polymorphisms classified as low-penetrance risk alleles for breast cancer susceptibility. Collectively, these variants account for approximately 18% of breast cancer heritability. Identifying shared risk alleles can enhance population-based risk stratification and improve genetic risk prediction.^98^ Nonetheless, most individual common risk alleles contribute only marginally to disease risk. The identification of hundreds of common risk alleles could pave the way for novel therapeutic strategies, including elucidating molecular pathways involved in breast cancer initiation and progression, developing innovative treatment approaches based on genetic susceptibility, and personalizing patient care by leveraging GWAS risk variants to predict drug response and optimize treatment plans.^97^

### 3.3. Updated genetic insights

Although numerous gene mutations have been found to be associated with hereditary breast cancer, a substantial number of hereditary breast cancer cases remain unexplained, as they lack mutations in known predisposition genes. To address this gap, recent studies have employed innovative genetic testing methods to identify novel genes whose mutations may increase breast cancer risk. For example, a study conducted in Jerusalem investigated 12 families with a high incidence of breast cancer, whose members tested negative for all known breast cancer predisposition genes.^113^ The researchers identified 70 genes previously unknown to be linked to breast cancer. The study employed a combination of cutting-edge machine-learning techniques and detailed protein structure analyses to investigate rare genetic variants. Notably, they discovered eight genes with candidate pathogenic variants linked to peroxisomal-related mechanisms in seven of the 12 families, suggesting that peroxisomal dysfunction may play an important role in breast cancer predisposition.

More recently, a large multi-ancestry fine-mapping study analyzed genotype data from 414, 746 females of African, Asian, and European ancestry to identify potential causal variants and putative target genes. The study uncovered a large number of association signals and candidate susceptibility genes for breast cancer.^114^ The researchers reported 332 independent association signals for breast cancer risk, with putative target genes enriched in key signaling pathways, including PI3K/AKT, TNF/NF-κB, and p53, which are involved in various cellular processes, such as metabolism, proliferation, apoptosis, tumor suppression, and inflammation.

These findings are broadening the horizon of the genetic research on breast cancer.

## 4. Screening

### 4.1. Early detection

For individuals with a family history of breast cancer, genetic testing is a dependable method for early detection. Additional screening techniques include MAM, clinical breast examination, and breast self-examination.^115^ Emerging biomarkers, such as miRNA levels, circulating cell-free tumor DNA, and circulating tumor markers, may also aid in early detection. Imaging techniques, such as MRI, MAM, and ultrasound, have been shown to reduce mortality and improve long-term survival by facilitating early tumor detection. In high-risk individuals, prophylactic surgeries represent a preventive intervention that may significantly lower the risk of developing breast cancer.^116^

### 4.2. Imaging

Digital MAM is considered the gold standard for breast cancer screening, with an estimated specificity of 99% and sensitivity of 78%. However, breast density affects sensitivity and specificity, particularly in women with heterogeneously dense or extremely dense breast tissue.

Digital breast tomosynthesis (DBT), also known as 3D MAM, improves imaging resolution by allowing for visualization of specific breast planes while minimizing tissue overlap. DBT enhances cancer detection in women with heterogeneously dense breasts or scattered fibroglandular densities but does not significantly improve detection in women with extremely fatty or highly dense breasts. In addition, DBT reduces recall rates across all breast density categories.^117^

Breast MRI is more sensitive than MAM or ultrasound, particularly in high-risk individuals, increasing the likelihood of early cancer detection. Prospective data indicate that, for every 1,000 women at higher-than-average risk who undergo additional physician-performed screening, 4.2 additional tumors are detected. This suggests that whole-breast ultrasound may improve incremental cancer detection rates in high-risk women. Studies indicate that, for every 1,000 women who undergo an automated whole-breast ultrasound, an additional 1.9 malignancies are detected.^118^

Whole-breast ultrasound is a viable alternative when breast MRI is not feasible, such as in cases where MRI costs are exorbitant, the patient is pregnant, has claustrophobia or severe anxiety, is in renal failure, or has a gadolinium allergy. However, breast MRI remains the most sensitive imaging modality for high-risk individuals.^119^

### 4.3. Screening recommendations

#### 4.3.1. Women at average risk

The majority of guidelines recommend mammographic screening for average-risk individuals aged 40 – 74 years, with women aged 50 – 69 being the optimal age group for screening. While no universal upper age limit is specified for breast cancer screening, some guidelines suggest that the decision to discontinue screening should be based on a woman’s overall health status. MAM is recommended as the primary screening modality for average-risk women, with most guidelines advising annual or biennial screening. Screening intervals should be determined on the basis of age, as recommended by some guidelines.^120^

Recommendations for clinical breast examination and ultrasound detection vary. The National Comprehensive Cancer Network and the American College of Obstetricians and Gynecologists recommend clinical breast examination every 1 – 3 years for women aged 25 – 39 years and annually for women of 40 years and older. However, all guidelines advise against the routine use of breast self-examination, MRI, and computed tomography for average-risk women due to insufficient evidence of benefit.^121^

#### 4.3.2. Women at higher risk

Breast cancer risk factors are categorized into five groups: (i) personal history of precancerous lesions and/or breast cancer, (ii) family history of breast cancer, (iii) known genetic predisposition to breast cancer, (iv) history of mantle or chest radiation therapy, and (v) dense breast tissue. For women at higher risk, annual MAM or MRI screening is recommended, with initiation at an earlier age than for average-risk individuals.

Women with biopsy-confirmed lobular carcinoma *in situ*, atypical ductal hyperplasia, ductal carcinoma *in situ*, or invasive breast cancer should undergo annual MAM or MRI screening following diagnosis.^120^ For those with a family history of breast cancer, annual MAM or MRI screening should begin 10 years before the age at diagnosis of the youngest affected relative, but not before the age of 30.^122^ Women with *BRCA1* or *BRCA2* mutations should start annual MAM or MRI screening at 25 – 30 years old. For those with a history of mantle or chest radiation therapy, regular screening should commence 8 – 10 years after radiation exposure.^123^

## 5. Conclusion

This review underscores the complexity of breast cancer by highlighting its diverse risk factors, genetic predispositions, and screening recommendations, all being essential to advancing knowledge, optimizing treatment strategies, and improving the quality of life of patients and survivors. Breast cancer oncogenesis is influenced by multiple factors, and its various histological subtypes are associated with distinct risk profiles. Given this complexity, future studies should focus on identifying effective risk reduction strategies, including sustainable lifestyle modifications and chemopreventive interventions tailored to each histological subtype. When combined with cutting-edge technologies such as molecular testing and MRI, MAM remains a cornerstone of early breast cancer detection, facilitating timely intervention and potentially improving patient outcomes. Ongoing research is crucial for refining screening techniques and developing tailored treatments. In addition, increasing public awareness, expanding access to screening, and fostering international collaboration among researchers and medical professionals are critical components in the continuous fight against breast cancer.

## Data Availability

Not applicable.
